# The Role of Dexmedetomidine in Pediatric Patients Presenting with an Anticholinergic Toxidrome

**DOI:** 10.1155/2021/7590960

**Published:** 2021-08-28

**Authors:** Mitchell Zekhtser, Erin Carroll, Molly Boyd, Shashikanth Ambati

**Affiliations:** ^1^Department of Emergency Medicine, Albany Medical Center, Albany, New York, USA; ^2^Department of Pediatrics, Massachusetts General Hospital for Children, Boston, Massachusetts, USA; ^3^Division of Pediatric Critical Care Medicine and Department of Pediatrics, Albany Medical Center, Albany, New York, USA

## Abstract

**Background:**

We report two pediatric cases of anticholinergic toxidrome, including the youngest reported to date, in which standard therapeutic strategies were either contraindicated or ineffective, while treatment with dexmedetomidine was rapidly efficacious with no adverse effects. Moreover, with the recent shortage of physostigmine, we highlight an alternative treatment in this clinical setting. *Case Summaries*. In case 1, a two-year-old had an overdose presenting with an anticholinergic toxidrome. However, his hypopnea precluded the use of benzodiazepines due to the high likelihood of intubation. In case 2, a 14-year-old had a polypharmacy overdose inducing agitated delirium that was refractory to high-dose benzodiazepines. Due to the unknown ingestion, physostigmine was avoided. In both cases, dexmedetomidine helped the patient remain calm and metabolize the ingestions.

**Conclusion:**

Our experience suggests that dexmedetomidine may be a useful adjunct in anticholinergic presentations in the setting of polypharmacy, when standard therapy is proven ineffective, contraindicated, or unavailable.

## 1. Introduction

Anticholinergic agents are ubiquitous and consistently rank among the top causes of pediatric toxicologic presentations. In the latest report by National Poison Data Systems, there are more than 40,000 exposures to antihistamines in children with exposures growing by over 1,000 every year over the last ten years. In 2018, antihistamines were found to be the second most common cause of pharmaceutical death in ages 13-19. This is in addition to other medications that cause anticholinergic presentations such as antipsychotics and nonpharmaceutical exposures [1]. Anticholinergic toxidrome results from competitive antagonism of acetylcholine at central and peripheral muscarinic receptors manifesting as tachycardia, anhidrosis, hyperthermia, mydriasis, agitation, delirium, seizures, and urinary retention. Management is largely supportive, with the mainstay of treatment being benzodiazepines for agitation and seizures. Physostigmine is generally reserved for more severe central and peripheral antimuscarinic symptoms [2]. Benzodiazepines potentiate GABA activity while physostigmine overcomes competitive acetylcholine inhibition as an anticholinesterase. Unfortunately, benzodiazepines effectively manage agitation in less than 24% of patients and confer risk of significant respiratory depression and worsening delirium [2, 3]. While recent articles argue that physostigmine is a safe and effective treatment of pure anticholinergic toxidrome [4], clinical use remains controversial and limited [5]. Many clinicians are hesitant to use physostigmine due to lack of familiarity and risk of potentially life-threatening side effects [6] which include arrhythmias, seizures, and airway compromise [7]. The risk of deleterious effects from benzodiazepines and/or physostigmine administration is enhanced in the setting of a clinical presentation that is clouded by polypharmacy. Thus, safe alternative therapies are needed. Though there are few case reports pointing toward dexmedetomidine's utility in anticholinergic and more complex mixed presentations, literature is limited in pediatrics to support its role in ingestions with suspected anticholinergic toxidrome [2, 8–12]. We report two cases of suspected polysubstance overdose presenting with primary anticholinergic side effects in which traditional therapeutic strategies were either contraindicated or ineffective, while treatment with dexmedetomidine was rapidly efficacious with no adverse effects. Informed consent was obtained from the guardian of the patient of cases one and two.

## 2. Case 1

**Figure 1:**
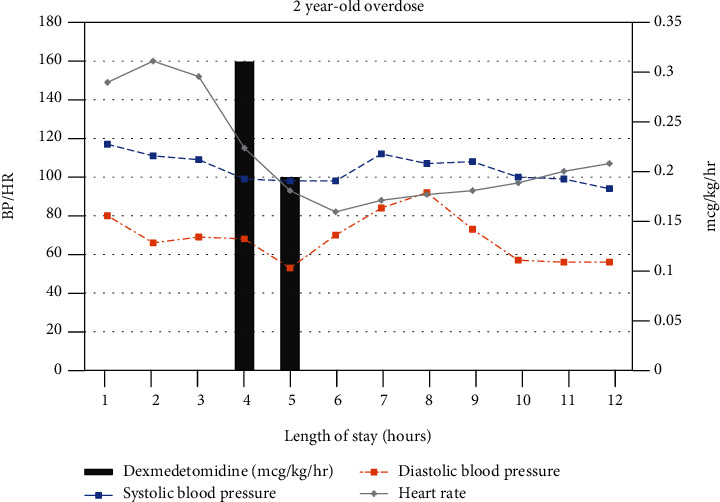
The patient is initially hypertensive and tachycardic. At hour 4, dexmedetomidine was introduced and the patient's vital signs began to improve.

An otherwise healthy 2-year-old male presented to our emergency department (ED) following an unwitnessed Dimetapp ingestion of unknown amount and formulation (common Dimetapp formulations: brompheniramine, dextromethorphan, and phenylephrine with or without acetaminophen or the nighttime formulation: diphenhydramine, phenylephrine). On exam, he was tachycardic and hypertensive with dry mucous membranes consistent with an anticholinergic crisis, requiring fluid resuscitation and benzodiazepines, thus necessitating admission to the Pediatric Intensive Care Unit (PICU). On arrival to PICU, he became hypopneic and lethargic, with a Glasgow Coma Score (GCS) of 9, and was started on high-flow nasal cannula. Electrocardiogram (EKG) showed a normal QRS and QTc interval. Laboratory evaluation showed no electrolyte abnormalities. Urine and serum drug screens were negative. Given his tenuous respiratory status, a dexmedetomidine infusion was initiated instead of using benzodiazepines to avoid potentiating further respiratory compromise with possible intubation. Dexmedetomidine was administered at 0.3 mcg/kg/hr for the first hour then decreased to 0.2 mcg/kg/hr the second hour, before being discontinued. The patient's heart rate and blood pressure appropriately decreased (Figure 1). He remained hemodynamically stable while maintaining his airway and was subsequently discharged home uneventfully.

## 3. Case 2

**Figure 2:**
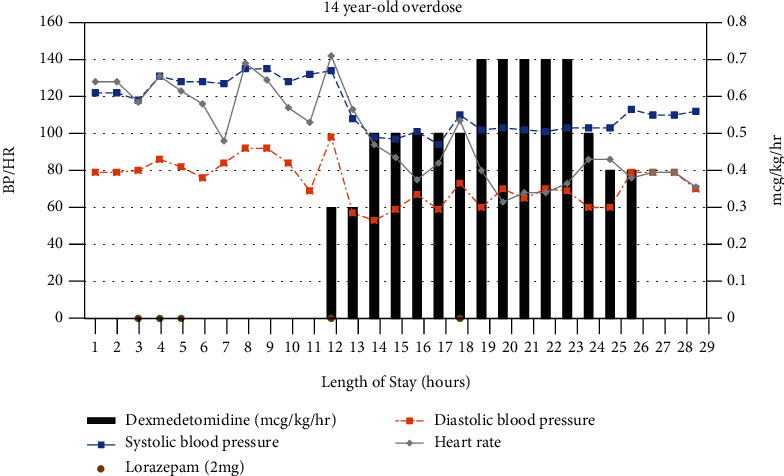
The patient had persistent tachycardia and hypertension despite multiple doses of lorazepam noted at hours 3-5. Dexmedetomidine was initiated at hour 12, and the patient had a significant improvement in vitals by hour 14 with mild increase in dose.

An otherwise healthy 14-year-old girl was transferred from an outside hospital after an unwitnessed intentional overdose involving over 100 diphenhydramine (25 mg) tablets with suspicion of other unknown over-the-counter medication ingestion. The patient was noted to have episodes of emesis and urinary incontinence. Labs were notable for presence of cannabinoids, mild transaminitis, acetaminophen level of 33, hypokalemia (2.9 mEq/L), and hypomagnesemia (1.6 mg/dL). EKG showed a prolonged QTc of 545 ms which later improved to 454 ms. At the outside hospital, the patient received 10 mg of lorazepam and another 6 mg in our ED due to persistent agitation, tachycardia, and hypertension before being transferred to the PICU. Her exam was notable for mydriasis, dry skin, hallucinations, agitation, and nonsensical speech despite multiple doses of lorazepam. Due to her persistent anticholinergic crisis, the decision was made to start her on dexmedetomidine. The team refrained from using physostigmine due to concerns of possible cardiotoxicity in the setting of QTc interval abnormalities and possible polypharmacy. She was started on dexmedetomidine at a dose of 0.3 mcg/kg/hr and slowly titrated to 0.7 mcg/kg/hr, which steadily improved her agitation and brought an appropriate resolution of her tachycardia and hypertension (Figure 2). Anticholinergic symptoms completely resolved within 13 hours allowing the patient to be cleared for discharge to an inpatient psychiatric facility.

## 4. Discussion

Anticholinergic agents block physiologic responses to acetylcholine via competitive antagonism at muscarinic receptors. The resultant parasympathetic blockade produces symptomatology like other hyperadrenergic states and toxidromes. For example, sympathomimetic toxidrome and serotonin syndrome also present with tachycardia, hypertension, mydriasis, and altered mental status. Furthermore, the distinguishing features of toxidromes (dry skin vs. diaphoresis, pupil size, urinary retention, and neuromuscular symptoms) may be obscured by polypharmacy [13, 14]. The two cases presented here illustrate the clinical heterogeneity and complexity introduced by toxic coingestions. Regardless of the causal agent, tachycardia, hypertension, and agitation with delirium remain priorities in clinical management. Dexmedetomidine's unique pharmacology makes it well suited to manage these physiologic derangements safely and effectively. Dexmedetomidine is a highly selective alpha-2 adrenoceptor agonist with inhibitory action on the locus coeruleus, the major noradrenergic nucleus of the brain, conferring its sympatholytic, anxiolytic, and sedating effects [15]. It is unique amongst sedatives in that it has minimal respiratory effects and induces an arousable sedation. These features, along with relative contraindications to benzodiazepines and physostigmine, made it the preferred treatment choice in both cases.

Hemodynamic derangements in anticholinergic toxidrome are largely a consequence of unopposed sympathetic activity and resultant disruption of many cardiovascular homeostatic regulatory mechanisms. Blunting of physiologic parasympathetic antagonism (mediated by cardiac M2 receptors) has a net chronotropic and dromotropic effect, resulting in resting sinus tachycardia. Similarly, hypertension results from the relative hyperadrenergic state, which is further exacerbated by agitation. Dexmedetomidine has been shown to decrease circulating catecholamines by 60-80% with predictable hemodynamic consequences. Reduction in heart rate and blood pressure should be considered an expected physiologic response to the sympatholytic effect that predominates at low doses [15]. Deleterious hemodynamic effects (e.g., bradycardia or hypotension) are dose-dependent and thus largely avoidable. Reported episodes are rarely of clinical significance and highly responsive to dose titration and fluid administration [10, 16]. This represents a significant advantage over physostigmine, which frequently requires multiple doses and is not as easily titratable [17].

An additional advantage of dexmedetomidine over physostigmine is its lower arrhythmogenic potential [18–20]. It is safe and may even be protective, in the setting of prolonged QTc [21–23]. This was particularly important in our second case, but relevant to both since diphenhydramine was one of the suspected agents in both patients. Diphenhydramine toxicity is dose-dependent with variation in potential ECG changes. Inhibition of fast sodium channels may lead to QRS widening, while potassium channel inhibition at higher doses can produce QT interval prolongation and abnormal ventricular repolarization [24–26].

The delirium classically seen in anticholinergic toxidrome is typically attributed to reduced acetylcholine activity at central muscarinic receptors [2]. However, advances in our understanding of delirium suggest that while decreased acetylcholine action may be the precipitant, it is likely only a part of a complex cascade of neuromodulatory events that perpetuates the state. Leading theories of delirium include neural dysconnectivity, various neurotransmitter hypotheses, neuroinflammatory, neuroendocrine, and diurnal dysregulation. Experts acknowledge that the true pathophysiology likely involves interplay amongst several of these mechanisms rather than one cause [27]. Dexmedetomidine is believed to exert neuroprotective effects possibly via central anti-inflammatory influences, downregulation of excitatory and upregulation of inhibitory neurotransmitters, or inhibition of oxidative stress [28]. Thus, there are many potential mechanisms, spanning the gamut of leading delirium theories, by which dexmedetomidine counteracts delirium. Conversely, physostigmine only addresses the deficiency of acetylcholine and thus may be insufficient in mixed toxicologic presentations. Lastly, benzodiazepines are known to be deliriogenic and can be associated with prolonging the PICU length of stay, while dexmedetomidine has been associated with decreasing the length of stay—partly due to its prevention of delirium [29].

## 5. Conclusion

Dexmedetomidine's utility in anticholinergic toxicity has been demonstrated in nine prior pediatric cases [8–10, 12]. The two cases we present support this growing literature and include the youngest case reported to date. Comparing our case report to others, we had no episodes of hypotension or bradycardia due to our initial low starting dose, 0.3 mcg/kg/hr compared to 0.5-1 mcg/kg/hr in all cases reports for the exception of one, as well as the way that our team titrated dexmedetomidine. In our first case, dexmedetomidine was used for just two hours in order to stabilize the patient allowing for a swift discharge in the morning. Additionally, due to the presentation of polypharmacy in both cases, we highlight the versatility of dexmedetomidine in this clinical setting. In the first case, due to the patient's hypopnea, initiating benzodiazepines may have caused further respiratory comprise potentially precipitating an unnecessary intubation. In the second case, our patient's symptoms were refractory to benzodiazepines. Physostigmine was considered but would require great caution given a recent history of prolonged QTc, and it is not as easily titratable as dexmedetomidine. Moreover, with the current physostigmine shortage, this may not be an option even in cases with an appropriate safety profile. Although patient two demonstrated many classic features of anticholinergic intoxication, certain features argued against a pure anticholinergic presentation including urinary incontinence. Both patients benefitted from dexmedetomidine's versatility in managing toxic presentations, with previously reported applications including management of serotonin syndrome, cannabis induced delirium, cocaine, and other stimulants [11, 30–34].

Safety still needs to be established before dexmedetomidine can be routinely recommended. In cases of pure anticholinergic toxidrome, physostigmine may be preferable, and benzodiazepines remain the treatment of choice in the actively seizing patient. Additionally, appropriate patient selection and careful hemodynamic monitoring are critical to avoiding potential deleterious side effects, as the appropriate antidote for dexmedetomidine is atropine, an anticholinergic, which can reprecipitate the initial presentation. However, in a setting of clinicians' comfort level with using physostigmine and its current drug shortage, dexmedetomidine may be a reasonable alternative medication in anticholinergic toxidrome. Further research is needed to determine if dexmedetomidine has a primary or adjuvant role in management of these cases.

## Data Availability

All supporting data and references can be accessed through PubMed resources.

## Supplementary Materials

Supplementary Materials CARE (case report) guideline checklist.
