# Supplementary material for: The Role of Dexmedetomidine in Pediatric Patients Presenting with an Anticholinergic Toxidrome
Source: Case Rep Crit Care. 2021 Aug 28;2021:7590960. doi: 10.1155/2021/7590960 (PMC8419523; doi:10.1155/2021/7590960)
Supplement: Supplementary Materials — CARE (case report) guideline checklist. [file 7590960.f1.pdf]

CARE case report guidelines

CARE Checklist of information to include when writing a case report

(cc) BY-NC-ND

Topic	Item No	Checklist item description	Reported on Page Number/Line Number	Reported on Section/Paragraph
Title	1	The diagnosis or intervention of primary focus followed by the words “case report”	P2/ Line 1	
Key Words	2	2 to 5 key words that identify diagnoses or interventions in this case report, including "case report"	P2/ Line 9	
Abstract (no references)	3a	Introduction: What is unique about this case and what does it add to the scientific literature?	P2/ Lines 15-18	
	3b	Main symptoms and/or important clinical findings	P2/ Lines 22-24	
	3c	The main diagnoses, therapeutic interventions, and outcomes	P2/ Lines 25-26	
	3d	Conclusion—What is the main “take-away” lesson(s) from this case?	P3/ Lines 3-5	
Introduction	4	One or two paragraphs summarizing why this case is unique **(may include references)**		Introduction P1
Patient Information	5a	De-identified patient specific information	P4/ L 11 & P5/ L 3	
	5b	Primary concerns and symptoms of the patient	P4/ L 12-15 & P5/ L4-6	
	5c	Medical, family, and psycho-social history including relevant genetic information	N/A	
	5d	Relevant past interventions with outcomes	P4/ L 15-16 & P5/ L8-9	
Clinical Findings	6	Describe significant physical examination (PE) and important clinical findings	P4/ L 14-18 & P5/ L10-11	
Timeline	7	Historical and current information from this episode of care organized as a timeline		Case 1/P1; Case 2/P2
Diagnostic Assessment	8a	Diagnostic testing (such as PE, laboratory testing, imaging, surveys).	P4/L 14-19 & P5 L 6-11	
	8b	Diagnostic challenges (such as access to testing, financial, or cultural)	N/A	
	8c	Diagnosis (including other diagnoses considered)	P4/ L 15 & P5 L 11	
	8d	Prognosis (such as staging in oncology) where applicable	P4/ L 23-25 & P5/ L 15-17	
Therapeutic Intervention	9a	Types of therapeutic intervention (such as pharmacologic, surgical, preventive, self-care)	P4/L15,16,22. P5/L7,8,14	
	9b	Administration of therapeutic intervention (such as dosage, strength, duration)	P4/ L 22 & P5/ L 14	
	9c	Changes in therapeutic intervention (with rationale)	P4/ L 20-22 & P5/ L 11-14	

Follow-up and Outcomes	10a	Clinician and patient-assessed outcomes (if available)	P4/ L 23-24 & P5/ L 15-17	
	10b	Important follow-up diagnostic and other test results	P4/ L 23-25 & P5/ L 14-15	
	10c	Intervention adherence and tolerability (How was this assessed?)	P4/ L 23-25 & P5/ L 15-17	
	10d	Adverse and unanticipated events	None	
Discussion	11a	A scientific discussion of the strengths AND limitations associated with this case report		Conclusion P 1 & 2
	11b	Discussion of the relevant medical literature **with references**		Discussions P 1-4
	11c	The scientific rationale for any conclusions (including assessment of possible causes)		Conclusion P 1
	11d	The primary “take-away” lessons of this case report (without references) in a one paragraph conclusion	P8/ L 15-23	
Patient Perspective	12	The patient should share their perspective in one to two paragraphs on the treatment(s) they received	N/A	
Informed Consent	13	Did the patient give informed consent? Please provide if requested	**Yes** ☑	**No** ☐

Please leave this space alone as it will be supplemented by the editorial office when needed.
